# Focused Cardiac Ultrasound to Detect Pre-capillary Pulmonary Hypertension

**DOI:** 10.3389/fvets.2022.830275

**Published:** 2022-03-01

**Authors:** Aurélie Lyssens, Marine Lekane, Kris Gommeren, Anne-Christine Merveille

**Affiliations:** Department of Small Animal Clinical Sciences, Faculty of Veterinary Medicine, University of Liège, Liège, Belgium

**Keywords:** tricuspid regurgitation pressure gradient, respiratory, echocardiography, scoring, emergency

## Abstract

**Background:**

Early recognition of pre-capillary (PC) pulmonary hypertension (PH) benefits dogs, allowing earlier treatment and improving prognosis. The value of focused cardiac ultrasound (FCU) to diagnose PH and assess its severity has not been investigated yet.

**Hypothesis:**

A subjective 10-point FCU pulmonary hypertension score (PHS) allows diagnosis and assessment of severity of PCPH.

**Animals:**

This study involved fifty client-owned dogs.

**Methods:**

Dogs, recruited between September 2017 and February 2020, were classified into four categories (no, mild, moderate, and severe PH; C1 to C4, respectively). C1 and C2, and C3 and C4 were regrouped as group 1 and group 2, respectively. A blinded general practitioner assessed four FCU cineloops. Five echocardiographic parameters were subjectively scored, resulting in a total score of 0–10. Non-parametric tests compared global scores between categories and groups. A receiver operating characteristic (ROC) curve determined the cutoff value to differentiate group 1 and group 2. A gray zone approach allowed diagnosing or excluding moderate to severe PH with 90% certitude.

**Results:**

Global scores were significantly higher for C4 than for C1, C2, and C3. Global scores of G2 were significantly higher than G1. The ROC curve indicated a cutoff value of 5, discriminating group 1 from group 2 with a sensitivity of 77% and a specificity of 100%. A score of ≥5/10 allowed diagnosing moderate to severe PH with ≥90% certainty while a score of ≤2/10 excluded PH with ≥90% certainty.

**Conclusions and Clinical Significance:**

Moderate to severe PCPH can be accurately detected by non-cardiologists using a 10-point FCU PHS score.

## Introduction

Pre-capillary (PC) pulmonary hypertension (PH) is defined as increased pressure within the pulmonary arterial vasculature and complicates a wide range of respiratory and systemic diseases in dogs (1, 2). In veterinary medicine, PH is defined as absent (<30 mmHg), mild (30–50 mmHg), moderate (50–75 mmHg), or severe (>75 mmHg) based on estimated systolic pulmonary arterial pressure (PAP) derived from tricuspid regurgitation velocity using Doppler echocardiography (1, 3–5). Moderate to severe PCPH is typically associated with clinical signs and negatively impacts survival. Management of PH in dogs focuses on treating the underlying cause and on medical treatment, such as phosphodiesterase-5 inhibitors to ameliorate clinical signs and delay disease progression (2, 3, 6, 7).

Prompt recognition of PCPH might benefit affected dogs, particularly in moderately and severely affected patients, resulting in earlier initiation of therapy and possibly improving quality of life (6, 8).

Unfortunately, diagnosis of PH remains challenging in veterinary practice. Thoracic radiographs, available to most practitioners, lack sensitivity to detect even severe PH (3, 9, 10). The gold standard to diagnose PH is invasive pressure measurement using right heart catheterization, which is rarely available and may be associated with significant risk and hence is seldom performed in markedly affected veterinary patients (1, 6, 11–13). Doppler echocardiography is the standard non-invasive method to diagnose PCPH in veterinary patients (1, 3, 10). Pulmonary arterial pressure can be estimated and morphological changes may be assessed, allowing classification of disease severity (2, 3, 5). In dogs with PH, right ventricular (RV) size remodeling and/or systolic dysfunction, systolic flattening of the interventricular septum (IVS), main and right pulmonary artery (PA) enlargement, alterations in the pulmonary artery flow profile, increased right atrial (RA) size, and distension of caudal vena cava (CVC) have been described using Doppler echocardiography (1, 5, 14, 15). Doppler echocardiography requires advanced equipment and profound skills. Focused cardiac ultrasound (FCU), as an alternative, is an easy, fast, and time- and cost-effective method, often used in emergency settings, to subjectively assess chamber size and cardiac function (16–20).

In 2019, a study of Vientos-Plotts et al. on point-of-care ultrasound (POCUS) through the subxiphoid view demonstrated that CVC distention (>1 cm), gallbladder wall edema, and ascites were poorly sensitive parameters to detect moderate to severe PH in dogs (21). To the authors' knowledge, information is lacking regarding the sensitivity and specificity of other FCU right-sided echocardiographic parameters to assess for the presence and severity of pre-capillary PH.

We hypothesized that a 10-point FCU pulmonary hypertension score (PHS) differentiates dogs with various degrees of PCPH and accurately identifies patients with moderate to severe PCPH.

## Materials and Methods

### Animals

Medical records from the small animal veterinary teaching hospital of the University of Liège were reviewed for canine patients diagnosed with PCPH between September 2017 and February 2020. Dogs were included if four cineloops were recorded and if the final echocardiographic diagnosis was either “normal cardiac examination” or “PH of precapillary origin.” Dogs with concomitant left- or other right-sided cardiac diseases were excluded, with the exception of dogs with mild mitral valve disease having an end-systolic left atrium to aorta ratio (LA/Ao) below 1.5. Based on the authors' assumption, an LA/Ao below 1.5 is unlikely to be responsible for post-capillary PH.

The diagnosis of PCPH was made by a board-certified cardiologist based on the criteria defined below. Included dogs were subdivided into four groups according to Doppler echocardiographic evidence of PH: category 1 (C1; normal cardiac examination), category 2 (C2; mild PH), category 3 (C3; moderate PH), and category 4 (C4; severe PH). C1 and C2 were subsequently further grouped as group 1 (G1; normal or mild PH), while C3 and C4 were grouped as group 2 (G2; moderate to severe PH).

### Echocardiography

Transthoracic 2D echocardiography, m-mode echocardiography, and conventional Doppler echocardiography were performed by one board-certified veterinary cardiologist using two ultrasound units (a: GE Vivid I from September 2017 to July 2018 and b: GE vivid E95 from August 2018 to February 2020) equipped with 2.2–3.5 and 5.5–7.5 and 1.4–4.6 and 2.4–8.0 MHz phased-array transducers, respectively. Dogs were placed in right and left lateral recumbency, and a simultaneous one-lead echocardiogram was recorded. Standard right parasternal (long and short axis) and left apical parasternal views were performed. Cineloops were recorded with a duration of 3–5 cardiac cycles.

PCPH was diagnosed and categorized based on the presence of an elevated tricuspid regurgitation pressure gradient (TRPG: >30 mmHg for C2, >50 mmHg for C3, and >80 mmHg for C4) or an elevated pulmonic regurgitation pressure gradient (PRPG: >19.5 mmHg for C2, >25 mmHg for C3, and >35 mmHg for C4) and the presence of indirect evidence of PH. Indirect evidence of PH included RV hypertrophy, RV enlargement, interventricular septal flattening, an enlarged main pulmonary artery, an enlarged right pulmonary artery on right parasternal four-chamber view, asymmetric and/or notched pulmonary flow, right atrial enlargement, a distended caudal vena cava, and presence of right-sided congestive heart failure signs (pericardial, pleural, or abdominal effusion).

### Focused Cardiac Ultrasound

Four bi-dimensional cineloops recorded by the cardiologist during the right-parasternal echocardiographic exam were selected: the longitudinal four-chamber view, short-axis trans-ventricular view, short-axis transaortic view, and subxiphoid view focused on the caudal vena cava.

All cineloops were subsequently assessed off-line by a single, blinded, general practitioner after a 1-h theoretical session on the PH score system. PHS consisted of the evaluation of five right-sided echocardiographic parameters: assessment of right heart chamber sizes (RA and/or RV), RV hypertrophy, IVS flattening, PA remodeling, and the presence or absence of right-sided congestive signs. A score of 0 to 2 was assigned for each, resulting in a PHS global score between 0 and 10 (Table 1).

**Table 1:** Pulmonary hypertension scoring system (PHS) for each evaluated right-sided echocardiographic parameter.

**Parameter**	**Score 0**	**Score 1**	**Score 2**
RA/RV enlargement	No right heart enlargement	RA *or* RV enlargement	RA *and* RV enlargement
RV hypertrophy	No RV hypertrophy	RV free wall hypertrophy *or* Presence of prominent papillary muscles	RV free wall hypertrophy *and* Presence of prominent papillary muscles
IVS flattening	No IVS flattening	/	IVS flattening
PA enlargement	No PA enlargement	Pulmonary trunk enlargement *or* Right PA enlargement	Pulmonary trunk enlargement *and* Right PA enlargement
Right-sided congestive signs	Absence of effusion and a normal CVC	Presence of effusion *or* distended non-compliant CVC	Presence of effusion *and* distended non-compliant CVC

*RA, right atrium; RV, right ventricle; IVS, interventricular septum; PA, pulmonary artery; CVC, caudal vena cava*.

### Statistical Analysis

Statistical analysis was performed with commercially available software (Xlstat, Addinsoft). Continuous variables were reported as median and range (minimum and maximum). The Shapiro–Wilk test was applied to assess the normal distribution of continuous variables.

Comparison between categories was performed with the Kruskal–Wallis test. When significant differences were identified, *post-hoc* pairwise comparisons were performed using Dunn's test with Bonferroni corrections. The Mann–Whitney *U*-test was used to compare G1 and G2.

Receiver operating characteristic (ROC) curve analysis was established to determine the ideal cutoff values to predict moderate to severe PCPH and to apply the gray zone approach.

Association between categories and right-sided echocardiographic parameters was assessed by a chi-square test with the *p*-value set at 0.005 to correct for multiple tests.

## Results

### Studied Population

Thirty-five dogs with confirmed PCPH were included in the study. Five in C2, ten in C3, and twenty in C4, respectively. Fifteen dogs were included in C1 (normal cardiac exam) and acted as the control population. The C1 and C2 categories were further grouped as group G1. The 30 dogs that were classified into C3 and C4 were further grouped into group G2.

Terriers (*n* = 11), Chihuahuas (*n* = 7), French Bulldogs (*n* = 6), Shepherds (*n* = 5), and crossbreeds (*n* = 6) were the most represented breeds diagnosed with PH in this study, followed by Spaniels (*n* = 3), Pugs (*n* = 2), Retrievers (*n* = 2), Bordeaux dog (*n* = 1), Beauceron (*n* = 1), Greyhound (*n* = 1), Dachshund (*n* = 1), Shih Tzu (*n* = 1), Beagle (*n* = 1), Maltese dog (*n* = 1), Shar Pei (*n* = 1), and Poodle (*n* = 1).

The age, weight, clinical history, and abnormalities on physical examination of each dog are listed in Table 2. In group G2, the dogs commonly presented with tachypnea (*n* = 15) and dyspnea (*n* = 15). Twenty-one dogs tested positive for antibodies against *Angiostrongylus vasorum* (1 in C1; 1 in C2; 3 in C3; 16 in C4). Six dogs presented with signs of right-sided congestive heart failure, all of which were classified with severe PH (C4).

**Table 2:** Summary of age, weight, clinical history, abnormalities on physical examination, and tricuspid regurgitation pressure gradient after echocardiography.

	**C1 (*n* = 15)**	**C2 (*n* = 5)**	**C3 (*n* = 10)**	**C4 (*n* = 20)**
Age in years (median; range)	3.5; 0.5–13	12; 0.5–13	11.5; 6–15	10; 2–12
Weight in kg (median; range)	17.1; 5.3–49.6	20; 3.5–24.8	9,3; 3.2–20.2	9.4; 2.8–40
**Clinical history (number of dogs)**				
No history	9	0	0	1
Dyspnea	1	2	5	10
Tachypnea	1	2	1	1
Exercise intolerance	3	1	3	12
Cough	2	3	5	5
Syncope	2	0	0	8
Abdominal distention	0	0	0	4
**Abnormalities on physical examination (number of dogs)**				
No abnormalities	11	2	1	1
Heart murmur	3	0	3	9
Bradycardia	1	0	0	0
Tachycardia	0	0	1	9
Crackles	0	1	4	8
Cyanosis	0	1	2	3
Tachypnea	0	0	5	8
Ascites	0	0	0	4
Presence of right congestive heart failure (number of dogs)	0	0	0	6

According to the classification of PH in dogs by Reinero et al. (1), all dogs in category C2 had PH secondary to respiratory disease, hypoxia, or both. Diseases included idiopathic pulmonary fibrosis (*n* = 2), bronchopneumonia (*n* = 1), bronchomalacia (*n* = 1), and tracheal collapse (*n* = 1). C3 included dogs with PH secondary to respiratory disease (*n* = 6), pulmonary thromboembolism (*n* = 4), or parasitic disease (*n* = 2). In C4, eight dogs were diagnosed with PH secondary to pulmonary thromboembolism, six dogs were diagnosed with idiopathic PH, five dogs had PH secondary to parasitic disease, and one dog had PH due to respiratory disease.

No statistical analyses were performed to compare the incidence between groups and categories for the abovementioned clinical signs and final diagnosis.

Before presentation to the cardiology service, 80% of the dogs in C2 received a treatment compared to 60% in C3 and 50% in C4. The different types of treatment used in each category are listed in Table 3.

**Table 3:** Types of treatment received before realization of an echocardiography.

**Treatment received before presentation**	**C2 (number of dogs)**	**C3 (number of dogs)**	**C4 (number of dogs)**
No treatment	1	4	10
Sildenafil	2	4	4
Benazepril	1	0	
Benazepil + furosemide	1	0	1
Benazepril + pimobendan	0	1	0
Digoxin + pimobendan	0	1	0
Pimobendan + furosemide	0	0	1
Pimobendan + sildenafil	0	0	1
Pimobendan + sildenafil + furosemide	0	0	1
Sildenafil + pimobendan + benazepril + spironolactone	0	0	1
Pimobendan + sildenafil + clopidogrel + aspirin	0	0	1

### PHS Score

**Figure 1:**
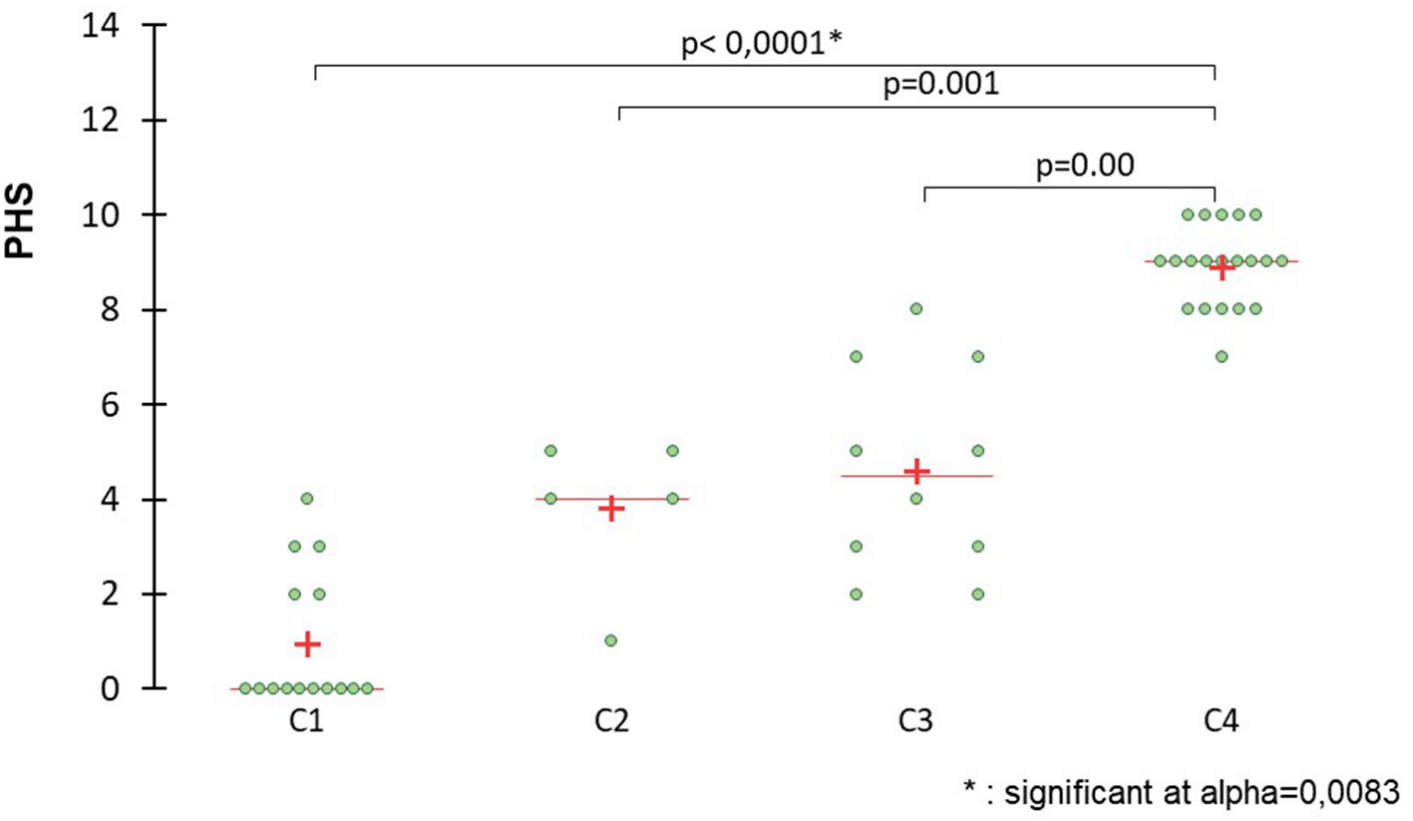
Scatter plots comparing global scores between C1 (control group), C2 (mild PCPH), C3 (moderate PCPH), and C4 (severe PCPH) to detect PCPH. The pulmonary hypertension (PHS) score is significantly higher for C4 than for C1, C2, and C3. The PHS for C3 is significantly higher than G1. The cross indicates the mean value and the horizontal line represents the median value.

**Figure 2:**
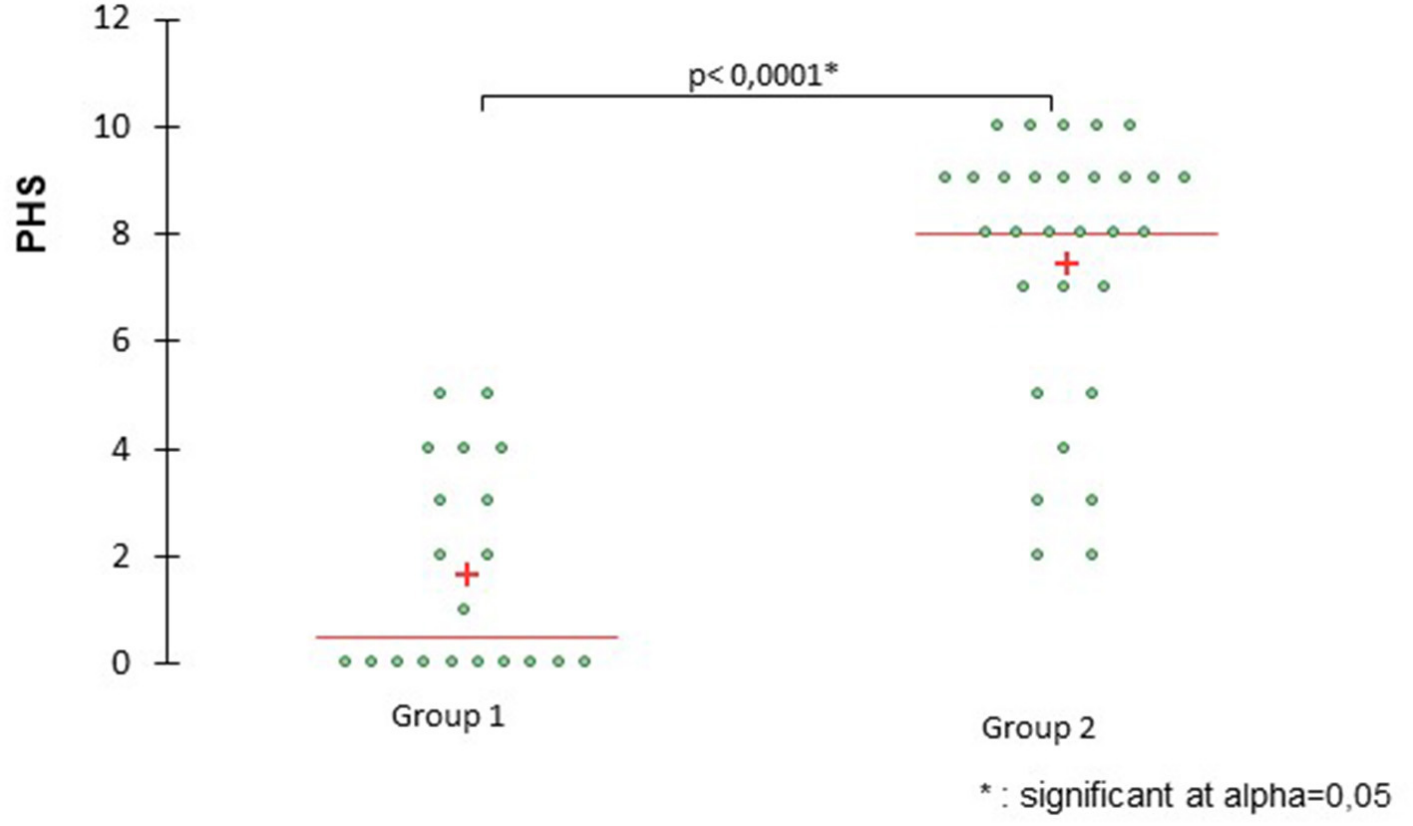
Scatter plots comparing global scores between group G1 (control group, C1 + mild PCPH, C2) and group G2 (moderate PCPH, C3 and severe PCPH, C4). The pulmonary hypertension score (PHS) for G2 is significantly higher than for G1. The cross indicates the mean value and the horizontal line represents the median value.

Global PHS scores, expressed as median and range, were significantly higher for C4 (9; 7–10) than for C1 (0; 0–4) (*p* < 0.001), C2 (4; 1–5) (*p* = 0.008), and C3 (4.5; 2–8) (*p* = 0.023). Global PHS score for C3 was significantly higher than for C1 (*p* = 0.023) but not for C2 (*p* = 0.84) (see Figure 1). Global PHS scores of G2 (8; 2–10 were significantly higher than G1 (0.5; 0–5) (*p* < 0.001) (see Figure 2).

**Figure 3:**
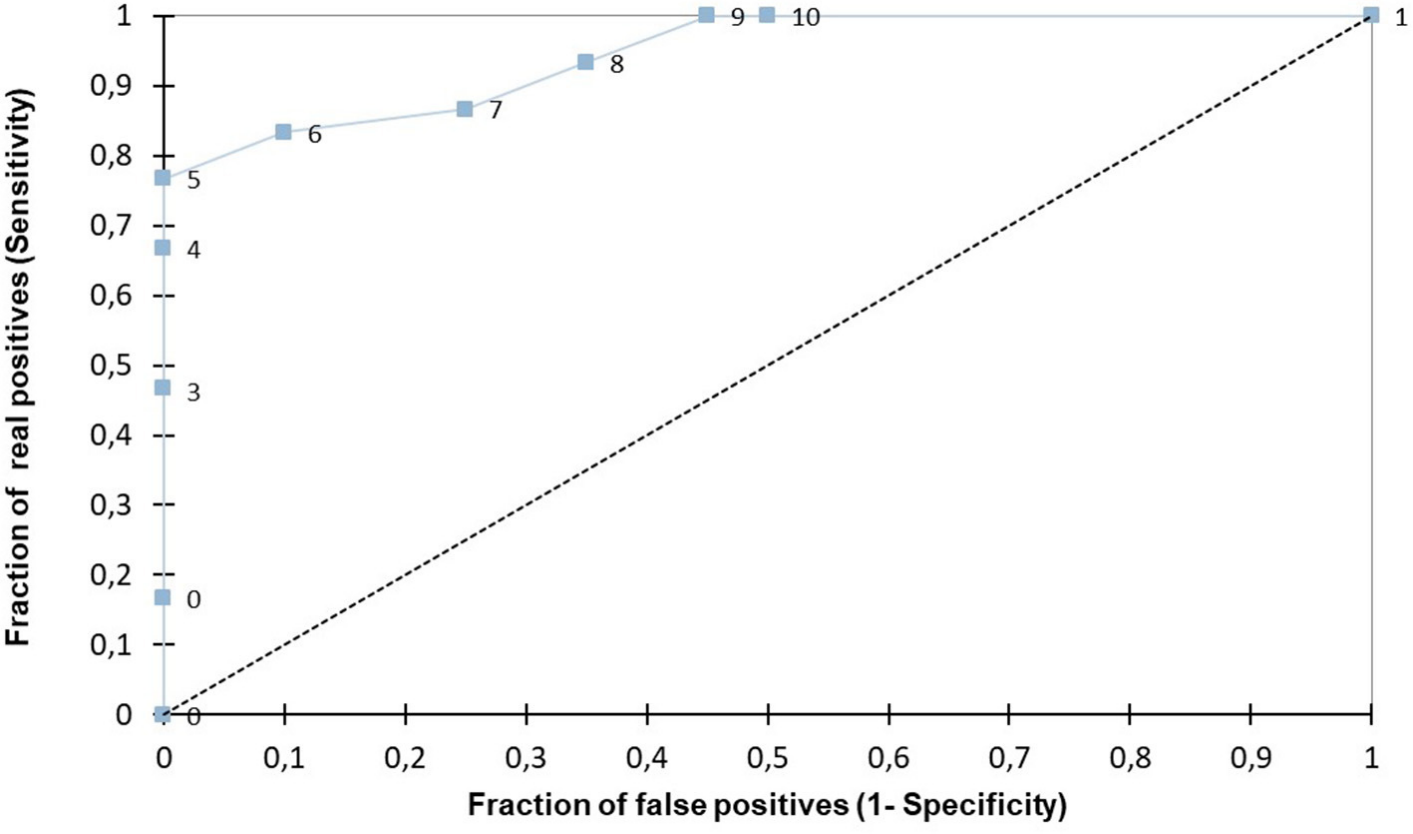
The area under the receiving operating characteristic (ROC) curve for the pulmonary hypertension score (PHS) indicates an optimal cutoff value of 5, discriminating G2 from G1 with a sensitivity of 77% and a specificity of 100% (AUC: 0.994; *p* < 0.001).

**Figure 4:**
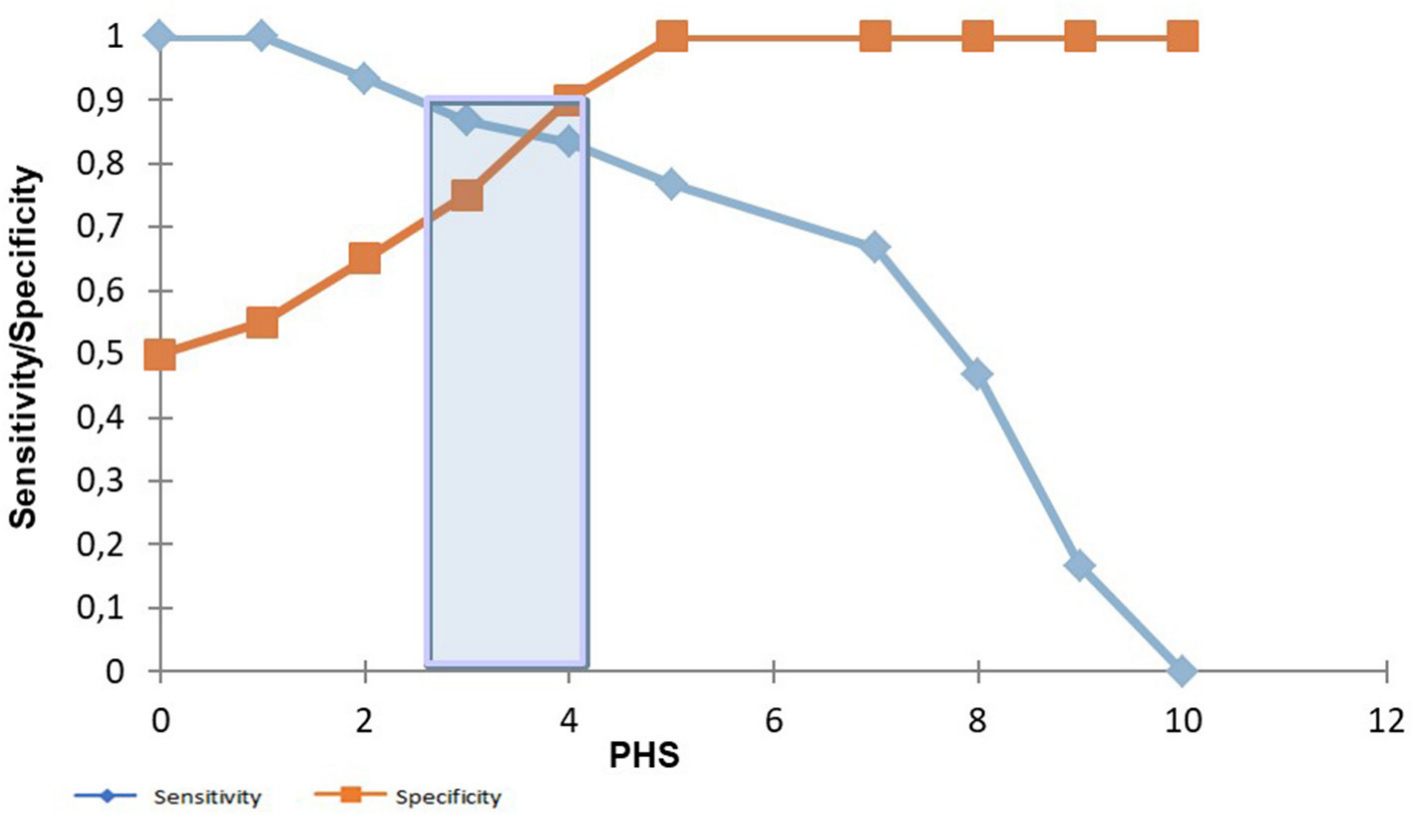
Comparison between sensitivity and specificity with the pulmonary hypertension score (PHS). A gray zone approach, presented by the blue bar. When a score of 5/10 is reached, a sensitivity of 77% and a specificity of 100% are reached to detect moderate to severe PCPH. When a score of 2/10 is obtained, a sensitivity of 93% and a specificity of 65% are reached to detect moderate to severe PCPH.

The area under the ROC curve for the 10-point score indicated an optimal cutoff value of 5, discriminating G2 from G1 with a sensitivity of 77% and a specificity of 100% (AUC: 0.944; *p* < 0.001) (see Figure 3). In Figure 4, a gray zone is presented. Based on the gray zone approach, a score of 5/10 or more allowed the clinician to diagnose moderate to severe PH with 100% certainty (sensitivity of 76.7% and specificity of 100%). A score of 2/10 or less excluded PH with more than 90% certainty (sensitivity of 93% and specificity of 65%).

Regarding the individual right-sided echocardiographic parameters, significant differences were found in the following right-sided echocardiographic parameters between C1, C2, C3, and C4: RV enlargement (*p* < 0.001), RA enlargement (*p* = 0.0013), RV hypertrophy (*p* < 0.001), prominent papillary muscles (*p* < 0.001), IVS flattening (*p* < 0.001), increased pulmonary trunk size (*p* < 0.001), and a subjectively large, non-collapsing CVC (*p* = 0.0018). Lower scores for these parameters were seen in C1 and higher scores were seen in C4.

## Discussion

This study demonstrated that moderate to severe PCPH can be detected with good accuracy by a non-cardiologist using a 10-point FCU PHS after 1 h of theoretical training. FCU is widely available in emergency settings and in general practice. It is fast, non-invasive, and cost-effective. It requires minimal training before use, as demonstrated in this study. Our results are promising to allow earlier detection of moderate to severe PCPH in dogs presenting an emergency with respiratory complaints, such as tachypnea, dyspnea, cough, exercise intolerance or syncope, and ascites. When a score of 5 out of 10 or more was obtained, it allowed us to state with 100% certainty that the patient had moderate to severe PCPH in this population. Using this score, treatment for PH can be started in emergency cases, while awaiting a complete echocardiography. When a score of 2 out of 10 or less is obtained, this allows the clinician to state with 90% certainty that the patient does not have moderate to severe PCPH; thus, treatment for PH should be avoided prior to further examinations.

This study showed that signs of RV enlargement, RA enlargement, RV wall hypertrophy, prominent papillary muscles, IVS flattening, and an increased pulmonary trunk size changed significantly in cases of PCPH and thus can be used to differentiate C1, C2, C3, and C4.

Two previous studies have evaluated the utility of right-sided echocardiographic parameters in the detection and differentiation of the different degrees of PCPH in dogs (5, 21). The study from Vientos-Plotts et al. (21) used only the subxiphoid view of the caudal vena cava as its right-sided FCU parameter. Vientos-Plotts et al. (21) did not find a significant difference in subjective CVC distention, CVC diameter, presence of gallbladder wall edema, or ascites between dogs with PCPH vs. the control group, nor between dogs with different degrees of PCPH. It was concluded that the caudal vena cava *via* the subxiphoid view should not be used as a sole screening test for the detection of moderate to severe PCPH in dogs.

In our study, signs of ascites and signs of a subjectively large and not collapsing CVC were included as signs of right-sided congestive heart disease. However, the presence of gallbladder edema or exact measurements of the diameter of the CVC were not assessed. Nevertheless, significant differences between C1, C2, C3, and C4 were present regarding the subjective assessment of the CVC (*p* = 0.0018). In accordance with the study of Vientos-Plotts et al. (21), there was no significant difference in the presence or absence of ascites between C1, C2, C3, and C4 (*p* = 0.006).

Beyond the CVC, our study included additional right-sided echocardiographic parameters that can be assessed during Doppler echocardiography while screening dogs for PCPH: RV remodeling, systolic IVS flattening, PA size, and RA enlargement. A previous study showed that enlargement of the RA, RV, and PA was common (88, 69, and 72%, respectively) in dogs with PH diagnosed by a complete echocardiography exam (5). These parameters may be clinically relevant to determine the prognosis (5). Our study, using FCU instead of complete echocardiography, confirmed the results published by Visser et al. (5). In our FCU, RA enlargement, RV enlargement, and PA enlargement could significantly (*p* < 0.001 for all 3 parameters) differentiate the four different categories of PH. The ability to differentiate between groups is expected to be useful for patient prognosis.

Identification of dogs with moderate to severe PCPH is of clinical significance. Dogs with moderate to severe PCPH require treatment and have a worse outcome than dogs with mild PCPH, in which treatment is not recommended (5). Earlier diagnosis in general practice or in emergency settings may allow for earlier treatment and possibly improve the outcome of the patient.

This study has several limitations. First, this study did not measure the PAP by right heart catheterization, which is the gold standard to diagnose PCPH in humans and dogs. Misclassification of normal dogs (C1) and of the degree of PH (C2, C3, and C4) was possible. Secondly, this study is a retrospective study and therefore lacked standardization of medications permitted. Forty percent of the dogs in C2, C3, and C4 were receiving a phosphodiesterase-5 inhibitor, sildenafil, before recording the cineloops. Sildenafil is a highly selective phosphodiesterase-5 inhibitor that causes nitric oxide-mediated vasodilatation and results in direct pulmonary artery vasodilatation and a reduction in pulmonary vascular resistance (2, 6). Different studies in veterinary medicine have investigated the consequences of sildenafil in dogs with PCPH (6, 22). Besides the use of sildenafil, some dogs in C2 and C4 also received diuretics before recording of the cineloops. In C2, one dog received a combination treatment of benazepril and furosemide. In C4, three dogs received furosemide and one dog was administered sildenafil before echocardiography. The administration of diuretics may alter the size of the CVC (23). The use of sildenafil and diuretics, such as furosemide and spironolactone, before recording the cineloops might have influenced the grouping of the dogs into the different categories and assessment by the cardiologist. Furthermore, all dogs were awake and breathing spontaneously during the echocardiography and thus during the recordings of the cineloops. In human medicine, it is reported that decreased chest wall compliance and, most importantly, the level of negative intrathoracic pressure influence caudal vena cava compliance (24–26).

Image acquisition varied during the study with the use of two different ultrasound machines, and the quality of the images could differ between the 50 animals. Additionally, pre-recorded cineloops were recorded by a board-certified veterinary cardiologist and with an echocardiograph of excellent quality. Less experienced clinicians and/or a less qualitative ultrasound machine might be encountered in general practice or in an emergency context. Variation between imagers could also occur, particularly between inexperienced imagers in the emergency setting; this study did not evaluate that.

To conclude, FCU may be a useful method to detect PCPH in dogs in general practice and in emergency settings. Moderate to severe PCPH can be detected with good accuracy on pre-recorded cineloops by non-cardiologists using a 10-point CV-POCUS score. A subjective 10-point CV-POCUS PHS score was significantly different between healthy and moderate-to-severe PH. Differences were also found between severely affected and healthy animals, and between mild and moderate PH. These findings require further investigation in first-line practice and in emergency settings.

## Data Availability Statement

The raw data supporting the conclusions of this article will be made available by the authors, without undue reservation.

## Ethics Statement

The animal study was reviewed and approved by Commission d'Ethique Animale nr° 18-2082. Written informed consent for participation was not obtained from the owners because we used images, obtained during echocardiography, of dogs that needed an echocardiography.

## Author Contributions

A-CM, KG, and AL contributed to conception and design of the study. A-CM organized the database and performed the statistical analysis. AL wrote the first draft and sections of the manuscript. All authors contributed to manuscript revision, read, and approved the submitted version.

## Conflict of Interest

The authors declare that the research was conducted in the absence of any commercial or financial relationships that could be construed as a potential conflict of interest.

## Publisher's Note

All claims expressed in this article are solely those of the authors and do not necessarily represent those of their affiliated organizations, or those of the publisher, the editors and the reviewers. Any product that may be evaluated in this article, or claim that may be made by its manufacturer, is not guaranteed or endorsed by the publisher.

## Abbreviations

CVC: caudal vena cava
FCU: focused cardiac ultrasound
IVS: interventricular septum
La/Ao: left atrium-to-aorta ratio
PA: pulmonary artery
PAP: pulmonary arterial pressure
PC: pre-capillary
PH: pulmonary hypertension
PHS: pulmonary hypertension score
POCUS: point-of-care ultrasound
PRPG: pulmonic regurgitation pressure gradient
RA: right atrium
ROC: receiver operating characteristic
RV: right ventricle
TRPG: tricuspid regurgitation pressure gradient.
